# Functional near-infrared spectroscopy studies in children

**DOI:** 10.1186/1751-0759-6-7

**Published:** 2012-03-20

**Authors:** Shinichiro Nagamitsu, Yushiro Yamashita, Hidetaka Tanaka, Toyojiro Matsuishi

**Affiliations:** 1 Department of Pediatrics, Child Health Kurume University School of Medicine, Kurume, Japan; 2 Department of Pediatrics, Osaka Medical College, Takatsuki, Japan; 3 Department of Pediatrics, Child Health, Kurume University School of Medicine, 67 Asahi-machi Kurume City, Fukuoka 830-0011, Japan

**Keywords:** Development, Children, Cognitive neuroscience, Near-infrared spectroscopy

## Abstract

Psychosomatic and developmental behavioral medicine in pediatrics has been the subject of significant recent attention, with infants, school-age children, and adolescents frequently presenting with psychosomatic, behavioral, and psychiatric symptoms. These may be a consequence of insecurity of attachment, reduced self-confidence, and peer -relationship conflicts during their developmental stages. Developmental cognitive neuroscience has revealed significant associations between specific brain lesions and particular cognitive dysfunctions. Thus, identifying the biological deficits underlying such cognitive dysfunction may provide new insights into therapeutic prospects for the management of those symptoms in children. Recent advances in noninvasive neuroimaging techniques, and especially functional near-infrared spectroscopy (NIRS), have contributed significant findings to the field of developmental cognitive neuroscience in pediatrics. We present here a comprehensive review of functional NIRS studies of children who have developed normally and of children with psychosomatic and behavioral disorders.

## Introduction

The medical management and treatment of children with psychosomatic, behavioral, and psychiatric disorders have gained recent attention among pediatricians because of the increased number of such patients visiting outpatient clinics. According to a nationwide epidemiological survey in Japan, 5.8% of all children who visited an outpatient pediatric clinic on a given day were considered to have a psychosomatic or psychosocial disorder [1]. As most pediatricians are not accustomed to examining these children, the Japanese Society of Psychosomatic Pediatrics has published clinical practice guidelines, which provide recommendations for the assessment, diagnosis, and treatment of pediatric psychosomatic disorders in primary care settings [2]. However, patient-reported outcome measurements may be required to establish clear evidence-based assessment of the effectiveness of these guidelines. Furthermore, better scientific understanding of the biological mechanisms underlying these disorders would highlight the significant associations between physical symptoms and the mind.

Developmental cognitive neuroscience in children is a rapidly growing research field that seeks to understand how the development of a child's brain is involved in the growth of the child's mind. In particular, advances in functional neuroimaging have revealed how localized cortical activity may be associated with behavioral responses during early human development and the impaired neural substrates in behavioral and cognitive dysfunction observed in child psychosomatic or psychiatric disorders. Over the past 20 years, functional magnetic resonance imaging (fMRI) technology has also greatly enhanced our understanding of developmental cognitive function in working memory and attention [3-5], and has revealed aberrant neural activation in adults with cognitive dysfunction such as depression, schizophrenia, and eating disorders [6,7]. This functional neuroimaging technique has also been applied to the understanding of developmental specialization for human voice processing, which plays a fundamental role in social communication. Dehaene-Lamberts et al. [8] discovered that when infants listen to speech in their native language, activation was not distributed widely, but was already concentrated to a set of left-hemispheric perisylvian regions, similar to the situation in human adults. In addition, the brain networks involved in processing non-speech human vocalizations, such as music, and emotionally stimulated vocalization, such as mother's voice, have also been studied using fMRI in early infant brain [9,10].

Near-infrared spectroscopy (NIRS) provides a new direction for developmental cognitive neuroscience research. The application of fMRI to infants is restricted to periods of sleep; however NIRS, a completely noninvasive neuroimaging technique, can be applied in the natural setting in children of any age, even whilst awake. This means that visual stimulation, such as viewing an emotional face, can be applied as a cognitive task while the subject is conscious. While there have been several recent reviews on functional NIRS for developmental cognitive neuroscience studies in adult neuropsychiatric disorders [11-13], there have been far fewer on clinical research in children. Due to its portability, NIRS is now being applied in all age groups including children and to various fields of cognitive science. Herein we review the breadth of functional NIRS studies of children who have developed normally and of children with psychosomatic or behavioral disorders.

## What is NIRS?

**Figure 1:**
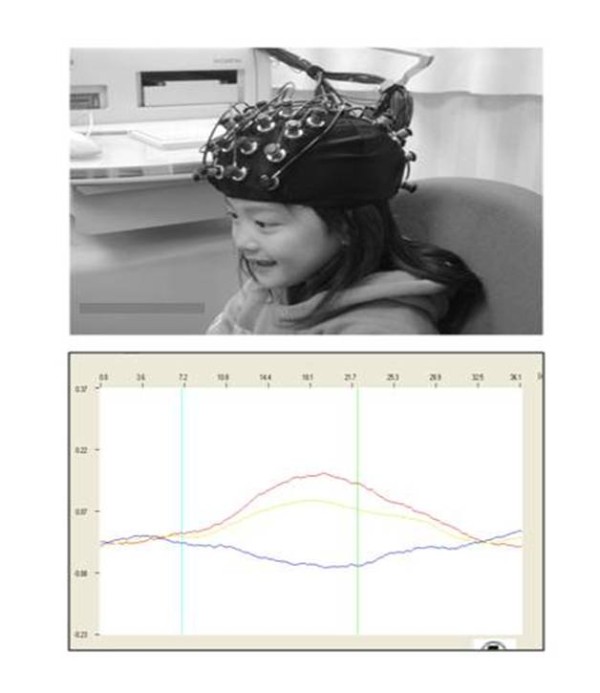
**NIRS apparatus, Hitachi Optical topography system ETG-4000 (Hitachi Medical Corp., Tokyo, Japan)**. The NIRS apparatus and ultrasound scanner were the same size. A head splint equipped with NIRS probes was attached (upper). Typical hemoglobin (Hb) response during a task (lower). The Hb concentrations are shown in red (oxy-Hb), yellow (total-Hb), and blue (deoxy-Hb). The blue and green vertical lines indicate the starting and ending times of a task, respectively.

Non-invasive NIRS has been used for more than a decade to examine hemodynamics and neural activation during various cognitive tasks by measuring concentration changes in oxy-, deoxy-, and total-hemoglobin (Hb). The basic principle of NIRS lies in different wavelengths of near infrared light being translated to changes in the concentrations of oxy- and deoxy-Hb. The general principles and technology of NIRS have been reviewed in several papers [14-17]. Brain activity involves regional changes in blood flow and oxygenation, based on the assumption that an increase in blood flow in turn increases the mean local oxygenation. It is known that the degree of increase in cerebral blood flow relatively exceeds the associated increase in regional cerebral oxygen metabolic rate, resulting in decreased deoxy-Hb in venous blood [18]. Thus, a typical hemodynamic response to cortical neuronal activation is an increase in total- and oxy-Hb, with a decrease in deoxy-Hb (Figure 1).

Compared to other neuroimaging techniques, NIRS has the advantages of complete noninvasiveness without requiring physical or mental restraints, a high temporal resolution of 0.1 s, and portable apparatus. Thus, it is suitable for imaging studies on children including newborns and infants, and can achieve detailed measurements of rapid changes in cerebral oxygenation during a task under natural conditions. NIRS has been mostly applied to the investigation of infant developmental cognitive function and perceptions including language, visual, olfactory, and auditory function and developmental changes of cerebral asymmetry associated with speech acquisition [19-23]. NIRS is also widely applicable for the investigation of infant-adult social interactions in infants [23-27]. Recently, psychiatric applications of NIRS to investigate frontal lobe function were reported in patients with depression, schizophrenia, and Alzheimer's disease [11,28]. The validity of NIRS for use in children is well established from previous studies into the developmental effects of child entertainment, psychiatric, and behavioral disorders [29-33]. The disadvantages of NIRS are 1) its low spatial resolution (20 mm), which makes it unsuitable for investigating the deeper parts of the brain, 2) NIRS measures hemoglobin concentration not only in the brain but also in other surface structures, such as the skin and skull, 3) the NIRS signal only reflects relative hemoglobin changes, not absolute values, and 4) there is no capacity for measuring brain structure for anatomical reference.

## When do infants recognize their mother's voice and face?

Functional NIRS studies have been applied to investigating early developmental cognitive function in newborns and infants. Indeed, reports on NIRS used to assess visual and auditory function in infants have increased in frequency recently. In particular, pediatricians are interested in the key developmental phase when infants clearly recognize their mother's face and voice. According to Pena's NIRS study [19], neonates showed larger neural activation in the left temporal area in response to forward speech than to backward speech and silence. They concluded that neonates are born with left hemisphere superiority to process specific properties of speech. Another NIRS study showed that frontal hemodynamic responses in infants during infant-directed speech (mother speaking to her baby slowly and with high pitch) by their own mothers were more activated than during speech by unfamiliar mothers [23]. This effect was significantly larger in infants aged 7-9 months compared to infants of other ages.

Similarly, the use of NIRS to investigate visual perception in infants is an increasing research area; specifically those stages when infants recognize and differentiate facial expressions such as happy and angry, frontal and profile views of faces, and upright and inverted faces. Based on characteristic time-course hemodynamic changes by NIRS recording, these visual perceptions in infants are acquired by 5-10 months of age [24-27]. Lloyd-Fox et al. [22] reported that infants as young as 5 months of age already have a specialized area in the bilateral temporal cortex for processing social dynamic stimuli and that this area was not activated by nonsocial dynamic stimuli. Further, presentation of a positive emotion on their mother's face evoked larger hemodynamic changes in the orbitofrontal cortex of infants, suggesting that these areas are important in the attachment process [27].

Similar NIRS studies have also been carried out in preschool children. Hoshi et al. [34] developed an NIRS apparatus combining a wireless telemetry system and a portable imaging system, and showed different hemodynamic patterns under various emotions, such as startled, anticipation, pleasant, and unpleasant, in 4- to 6-year-old preschool children. NIRS is thus a promising neuroimaging technique for understanding developmental cognitive function and interrogating infant-adult social interactions in early childhood.

## Are video games harmful for child brain development?

Much interest has been focused on the effects of video game playing on brain development and the development of cognitive ability in children. Many parents have expressed concerns about the amount of time their children spend on video games and about the relationship between this activity and adverse behavior. Such games have also been associated with many adverse effects, such as photosensitive epilepsy, enuresis, encopresis, visual injuries, obesity, tendonitis, hand-arm vibration syndrome, and enhancement of allergic responses [35-38]. Additionally, many studies have investigated the influences of video games on aggressive behavior in children [39]. On the other hand, familiarity with a wide range of video games can help reciprocal conversation ability in children, which may enhance sociability [40]. In particular, "electronic friendship" is often required among boys. Despite the great popularity of video games, there have been only a few studies examining the biological effects of video games on brain activity.

**Figure 2:**
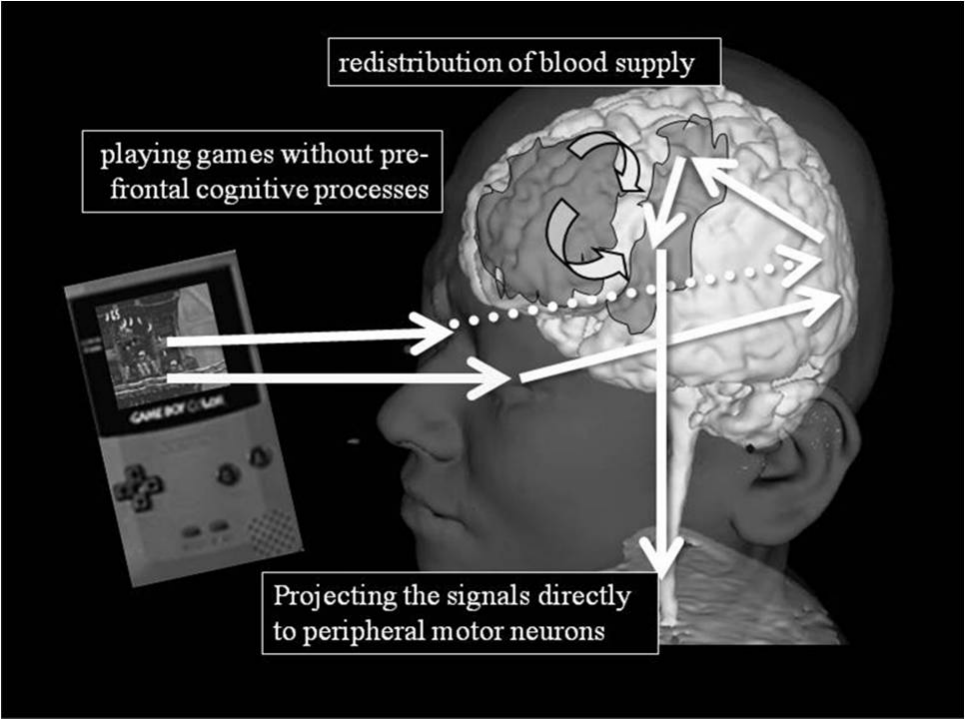
**Schematic image of neural signals in children while playing video games**. Neuronal activity in the occipital area caused by visual stimulation transmits and activates the motor cortex area without involving the prefrontal neuronal activities. Blood flow in the prefrontal area might be diverted into the motor cortex area (curved arrows), as neuronal activities around the motor cortex were activated more with frequent tapping on the game controller.

We previously measured cerebral Hb concentrations using NIRS in children and adults to investigate the effect of video game playing on regional cerebral blood flow [29]. Significant increases in bilateral prefrontal total-Hb concentrations were observed in some of the adults during video game playing, whereas significant decreases in bilateral prefrontal total-Hb concentrations were seen in some of the children. Moreover, a significant positive correlation between mean oxy-Hb changes in the prefrontal region and those in the bilateral motor cortex area was seen in adults, while the same measurement in children produced a significant negative correlation. As playing video games increases systolic and diastolic blood pressure, heart rate, and oxygen consumption, it is possible that high game performance (in other words, frequent tapping on the game controller, especially in children) leads to increased blood flow around the motor cortex area. As a result, blood flow in the prefrontal area might be diverted into the motor cortex area during video game playing. Neuronal signals would pass through the motor cortex after integrating perceptual stimulation at the visual association area, projecting the signals to peripheral motor neurons without passing through prefrontal neurons in children (Figure 2). In addition to the level of interest in the game, extra attention devoted to the task might contribute to the various changes in Hb concentrations. Matsuda et al. [30] also reported a sustained decrease in oxy-Hb during video game playing in the dorsal prefrontal cortex in children. They also mentioned that the more subjects increased their attention to visual stimuli, the more the Hb signal in the dorsal prefrontal cortex decreased. According to their interpretation, the negative correlation may be explained by an attenuation of higher common neural activity of prefrontal cortex neural activity at rest by paying attention to external stimuli.

Further prospective NIRS studies during video game tasks may reveal an age effect with respect to reduced hemoglobin concentration in the prefrontal area. Such studies in groups of different aged children could provide a hint as to the optimal usage of video games in childhood.

## Attachment perception overcomes body image in childhood anorexia nervosa

Anorexia nervosa (AN), which commonly occurs during adolescence in girls, is a disturbance of eating habits characterized by excessive preoccupation with body weight, shape, and food intake [41]. AN has also been reported in children, especially in the prepubertal age group. The risk of developing and preserving the symptoms of AN seems to be multifactorial, with individual, familial, sociocultural, and biological factors interacting within the developmental framework.

NIRS studies have been used to investigate cognitive patterns in AN. Uehara et al. [12] reported lower activation and a gradual increases in oxy-Hb and decreased deoxy-Hb concentrations in the prefrontal cortex during word-fluency tasks in adult AN subjects. These authors suggested that specific patterns of changes in oxygenation might indicate less supply and less demand for cerebral blood volume during cognitive tasks in AN. The clinical symptoms of AN, such as dieting tendency and eating behavior problems, showed negative correlations with regional hemodynamic changes in the frontotemporal and orbitofrontal cortex, respectively [42]. We also identified hemodynamic changes by NIRS during the word fluency task in children with AN (mean age, 14.2 years) [31]. The children with AN were consistently characterized by an unchanged or less fluctuating response pattern of total-, oxy-, and deoxy-Hb concentrations during the task and rest periods. On the other hand, the total- and oxy-Hb concentrations immediately increased and the deoxy-Hb concentration immediately decreased after the beginning of the task and gradually reached the baseline level after the end of the task in the control group. These different prefrontal hemodynamic responses might indicate that AN subjects apply fewer brain circuits or fewer neurons per circuit during cognitive tasks and might use different brain circuits in relation to their preoccupation with eating behaviors.

**Figure 3:**
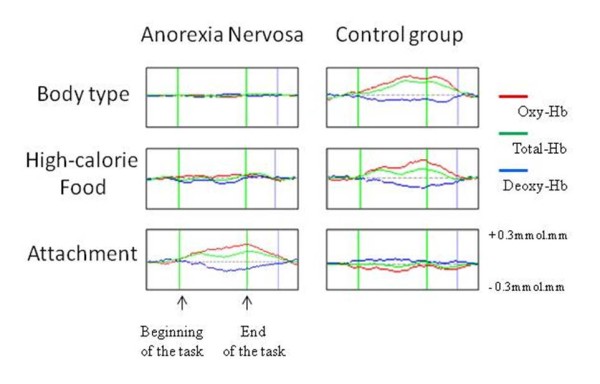
**Grand averages of the waveforms of each hemoglobin (Hb) change for all subjects (12 females with anorexia nervosa and 13 female controls) during each task**. A representative channel in the prefrontal area is presented. The increase in oxy-Hb was larger in the anorexia nervosa group than in the control group during the attachment task. Unchanged or less fluctuating response patterns of each Hb concentration are shown in the anorexia nervosa group when viewing body type of high-calorie foods.

NIRS studies might also be applicable to measuring hemodynamic responses during disorder-specific tasks such as viewing high-caloric food or different body types. Based on the implication of a disrupted attachment to the mother during early child development contributing to the development of AN [43], we applied a task of attachment between mother and child in addition to other disorder-specific tasks such as viewing high-caloric food or different body types [32]. Photographs in which a mother is talking delightedly with her daughter or a mother is kissing her baby were used. Interestingly, the childhood AN group tended to show smaller increases in prefrontal blood volume when viewing images of slender and obese body types and high-calorie food than the control group, but greater increases in prefrontal blood volume when viewing images of mother-child attachment than the control group (Figure 3). This result suggested that excessive concern and distorted perception regarding attachment might be critical in the pathogenesis of childhood AN and that reconstruction of the mother-child attachment process could play an important role in treating AN rather than working to reduce the excessive preoccupation with body weight, shape, and food intake.

## The restless child: NIRS research on ADHD

Attention deficit hyperactivity disorder (ADHD) is the most frequent neuropsychiatric disorder in children, and its incidence has increased in recent years. The estimated prevalence of ADHD is 3-7% [44]. Difficulty concentrating, hyperactivity, irritability, and impulsivity are the primary symptoms in children with ADHD, which may cause impairments in school performance, peer relationships, and self-esteem. In comparison with other imaging methods such as functional MRI, positron emission tomography, and single photon emission computed tomography, NIRS is relatively insensitive to movement artifacts and its inspection time is considerably short. Thus, even a restless child can successfully undergo imaging by NIRS.

Functional and anatomical alternations have been reported in the frontal cortex of adults with ADHD. Negoro et al. [33] thus investigated prefrontal hemodynamic responses in children with ADHD (mean age, 9.6 years) using the Stroop color-word task, a classical measure of frontal lobe function. The oxy-Hb changes in children with ADHD were significantly smaller in the bilateral inferior lateral frontal cortex than were those of the control group during the task. Jordan Moser et al. [45] also reported increased deoxy-Hb just after stimulation onset and delayed oxy-Hb increases in the right dorsolateral prefrontal cortex in children with ADHD using the Stroop task, indicating higher oxygen consumption and higher brain activation in that area during the task. Future NIRS studies could evaluate the effects of medications used to treat the ADHD symptoms based on hemodynamic response patterns.

## NIRS application for adult psychosomatic symptoms

Several research groups have reported on the usefulness of NIRS in investigating the association between frontal lobe dysfunction and social functioning in psychiatric disorders such as depression and schizophrenia. Suto et al. [11] demonstrated changes in the disease-specific pattern of prefrontal oxy-Hb during word fluency tasks in patients with these disorders. The depression group was characterized by a smaller oxy-Hb increase during the first half of the task period, and the schizophrenic group showed a small trough of oxy-Hb at the start of the task period and an oxy-Hb re-increase following the task period. Reduced activation in prefrontal oxygenation was also associated with lower self-assessment of social functioning.

NIRS has also been applied in psychosomatic medicine to evaluate mental stress-induced prefrontal cortex activity and its relationship with sympathetic nerve and hypothalamic-pituitary-adrenal (HPA) functioning under mental stress task conditions. Specifically, Tanida et al. [46] evaluated the relationship between skin condition and left/right asymmetry in the prefrontal cortex activity during mental stress tasks using NIRS. Subjects who exhibited right-dominant prefrontal cortex activity during mental stress tasks had higher levels of sebum secretion in the facial skin, suggesting that such subjects are sensitive to mental stress associated with hyperactivity of the HPA axis. They also reported improvement in the unilateral dominancy of stress-induced prefrontal cortex activity with reduced sebum secretion by exposure to fragrances [47].

Future prospects of NIRS application for psychosomatic disorders are promising. As biological evaluation of cognitive and autonomic nerve functions demonstrates the benefits of understanding the degree of psychosomatic symptoms and determining therapeutic efficacy, NIRS is rapidly becoming a valuable tool for psychometric measurement.

## Conclusion

This review demonstrates recent NIRS studies on children in the field of developmental cognitive neuroscience. NIRS enables us to effectively and noninvasively investigate developmental acquisition of an infant's visual and auditory function and child-mother attachment level. In particular, the difference in the hemodynamic effect of playing video games between children and adults was assessed, based on the popularity of this task in the preferred activities of children. The application of NIRS has also been extended to investigating cognitive dysfunction in child behavioral and psychiatric disorders such as ADHD and AN. NIRS shows promise for assessing the cognitive function of children and adults with psychosomatic disorders and is expected to be more widely used in the near future.

## Ethical approval

The design of our study and the procedures for obtaining informed consent were based on the principles of the Declaration of Helsinki, and approved by the Medical Ethics Committee of Kurume University School of Medicine. Informed consent was obtained from each child and his/her parents prior to the study.

## Competing interests

The authors declare that they have no competing interests.

## Authors' contributions

SN deigned the study and collected the data. YM and TM provided advice on the data analysis. SN drafted the manuscript. HT participated in revision of the manuscript. All authors have read and approved the final manuscript.
